# Neurosurgeons and the fight with COVID-19: a position statement from the EANS Individual Membership Committee

**DOI:** 10.1007/s00701-020-04360-3

**Published:** 2020-05-29

**Authors:** Mario Ganau, David Netuka, Marike Broekman, Cesare Zoia, Eleni Tsianaka, Michael Schwake, Naci Balak, Amitendu Sekhar, Sami Ridwan, Hans Clusmann, EANS IM Committee

**Affiliations:** 1 Department of Neurosurgery, Oxford University Hospitals NHS Foundation Trust, Oxford, UK; 2 Department of Neurosurgery and Neuro-oncology, 1st Medical Faculty, Charles University, Central Military Hospital, Prague, Czech Republic; 3 Department of Neurosurgery, Haaglanden Medical Center, The Hague, & Leiden University Medical Center, Leiden, The Netherlands; 4 Department of Neurology, Massachusetts General Hospital, Boston, MA USA; 5 Department of Neurosurgery, Fondazione IRCCS Policlinico San Matteo, Pavia, Italy; 6 Department of Neurosurgery, Dar Al Shifa Hospital, Kuwait City, Kuwait; 7 Department of Neurosurgery, University Hospital Muenster, Muenster, Germany; 8 Department of Neurosurgery, Istanbul Medeniyet University Göztepe Education and Research Hospital, Istanbul, Turkey; 9 Department of Neurosurgery, Paras Hospital, Udaipur, Rajasthan India; 10 Department of Neurosurgery, Paracelsus-Klinik, Osnabrück, Germany; 11 Department of Neurosurgery, RWTH Aachen University, Aachen, Germany

## Introduction

The growing SARS-CoV-2 pandemic is posing many new challenges to neurosurgeons, who have been forced to modify their working patterns by reducing/halting their elective theatres, triaging patients requiring emergency interventions, and helping out in other units such as emergency department and intensive care units. This outbreak is opening up entirely new scenarios; as such, concerns have been raised regarding the safety of patients and frontline healthcare professionals.

To address these novel challenges, the following aspects are of particular importance to individual neurosurgeons:

1. Lack of adequate information on SARS-Cov-2 and its disease (COVID-19)
2. Ambiguity regarding appropriate personal protection equipment (PPE)
3. Barriers to standard communication protocols
4. Acute and chronic psychological distress
5. Challenging the existing ethical fundaments

The European Association of Neurosurgical Societies (EANS) has an Individual Membership Committee (IMC) whose main goal is to represent all EANS individual members from all over the world, ensuring that their voices are heard and their concerns are brought up to the various areas of the Society. This is done by constantly collecting individual members’ wishes and their daily challenges, making sure that they are heard at all levels; as such, the IMC gathers multiple times a year, either face to face or through virtual meetings. To try and support as many neurosurgeons as best as we can, the IMC decided to draft this position statement with the following purposes: (a) defend neurosurgeons’ right to seek and obtain adequate protection equipment, (b) highlight the need for constant update through online educational sources, and (c) favour neurosurgeons’ access to psychological support when needed.

This statement was written by a task force created by IMC members; it references widely the latest evidence available in the international literature and leverages on the efforts already put in place by our Society through its website which is serving as a living platform for breaking news, discussing of hot topics and presenting agreement over best practices [2, 4, 12]. Finally, this statement covers the appropriate communication strategies to be implemented within neurosurgical teams at time of COVID-19 and outlines the need for a shared pan-European response to the crisis.

## Access to adequate information and importance of qualitative research

The scientific literature on the impact of COVID-19 on neurosurgery is utterly fragmented, characterised by small reports with good regional representativeness, but limited external validity [13, 17]. European experience on clinical courses of COVID-19 in general has been published recently [3]. For this reason, collecting relevant neurosurgical data, outcome metrics and frontline information is essential. To overcome such lack of knowledge, the IMC decided to keep a close eye on the many surveys that have been released recently in order to assess how neurosurgeons all over the world are responding to the outbreak, how their professional and personal life has been affected, and last but not the least, how they are feeling in the midst of the potentially worst pandemic since over a century. The IMC has closely worked with authors of each survey (see Table 1) to facilitate their distribution among the international neurosurgical community and increase their statistical significance through high participation rate. Surveys could certainly fill unmet needs, such as the lack of granular data about the pandemic or the cultural and practical reasons behind different approaches adopted in different countries. It is therefore reasonable to expect relevant answers from this type of qualitative studies.

**Table 1:** Open surveys on the impact of COVID-19 on neurosurgery

Geographical outreach (language)	Sponsor (website)	Hyperlink
Worldwide (English)	World Federation of Neurosurgical Societies – WFNS (https://www.wfns.org/)	https://it.surveymonkey.com/r/VKW7NFR
USA/worldwide (English)	Global BrainSurgery Initiative (https://www.gwmedicinehealth.com/departments/faculty/global-brainsurgery-initiative/)	https://surveymonkey.com/r/J5ZDJ6D
Europe/worldwide (English)	European Association of Neurosurgical Societies - EANS (https://www.eans.org)	https://www.surveymonkey.de/r/PG8GYGS
Worldwide (English)	AO Spine Foundation (https://aospine.foundation.org)	https://aospine.aofoundation.org/about-ao-spine/news/2020/2020_03-covid-survey
Iberian Peninsula and Latin America (Spanish)	Neurocirurgia Contemporánea (http://neurocirurgiacontemporanea.com)	https://forms.gle/BcMa2YDUob7PRVMx7
UK (English)	The Society of British Neurological Surgeons - SBNS, Neuro-Oncology Section (https://sbns.org.uk)	To be released soon through SBNS website
Australia/worldwide (multiple languages)	The Cochrane Collaboration	https://sydneypublichealth.au1.qualtrics.com/jfe/form/SV_1AmgErWpIJVSAYd
Worldwide (multiple languages)	Harvard, Oxford, Cambridge, Princeton, NYU, IESE, MIT, Boston and Warwick Universities	https://covid19-survey.org/

## Personal protection equipment

One greatest source of concern, at present, is the lack of agreement on what PPE should be adopted and in which circumstances (e.g. daily ward rounds of neurosurgical patients admitted to COVID-19 wards, involvement in aerosol-generating procedures on COVID-19 positive patients and those suspected to be so, etc.). This issue has been constantly revised by the national and international public health authorities, with several updates which resulted in very different attitudes across different countries and sometimes even among regions. Such heterogeneity might depend on the highly variable number of cases isolated in each country, which simply represent the different stages of COVID-19 spreading across the world. Whereas we acknowledge that possible shortages can occur in the next weeks/months, hospitals should plan and enforce strategies to ensure the protection of their staff at any time without exceptions. The latest recommendations from the World Health Organization (WHO), consistent with the evidence from systematic reviews, highlight that adequate PPE should be used, depending on the level of exposure. To this extent, Level 2 PPE, consisting of masks (US N95, EU FFP2 or equivalent), long sleeved disposable gown, gloves and eye protection, should be worn whenever assisting with any procedure generating a moderate amount of fluid, spray and debris [15]. On the contrary, for all aerosol-generating procedures in COVID-19 positive patients, Level 3 PPE consisting of full-face respirators or FFP3 masks with visors, double gown, double gloves and rubber boots is recommended [15]. Depending on the neurosurgical intervention, a moderate to higher amount of aerosol can be generated by craniotome cutters, high-speed drills, bipolar/monopolar cautery, and even rinsing of the surgical site. As such, appropriate training before accessing surgical theatres is highly advisable: all masks should be fit-tested and not just fit-checked, and a risk assessment should be conducted by the entire team (surgeons, anaesthetists, scrub nurses and operating department practitioners) to select the needed PPE. This process is meant to ensure protection of the entire staff from both droplets (which are short lasting around the surgical site) and microdroplets (which, once generated, can last for over 30 min in the environment) [1]. On the other hand, Level 1 PPE consisting of surgical mask, plastic apron and non-sterile gloves should be considered whenever engaging on low-risk close patient contact clinical activities, such as ward rounds, physical examination and diagnostic imaging [15]. Since several members reported that testing is not always possible at the time of patient admission, or before carrying out a surgical procedures, especially in a time when emergencies are more common than non-deferrable elective interventions, neurosurgeons should have access to adequate PPE for all cases, at all times, without exception. There are some preliminary reports suggesting that (low-dose) native CT thorax imaging may increase the rate of early detection of COVID-19 in our patients; however, in asymptomatic patients, the irradiation exposure is a matter of discussion [10]. Until antibody testing will be widely available, or any other biotechnology/nanomedicine-based point-of-care device introduced for rapid and sensitive detection of SARS-Cov-2 [6, 14], the use of adequate PPE is the only feasible one to avoid accidental contamination due to late recognition of patients’ positive status, which could largely jeopardise the safety of entire surgical theatres and wards.

## The importance of communication

COVID-19 crisis has got a severe impact on community hospitals and tertiary centres, their internal organisations, networks and referral systems. Several members suggested adopting a split teams ROTA (1 week on, 1 week off), so that teams never meet in person despite ensuring a continuity of care through virtual handovers, flexible on-calls and immediate support to those falling sick. In a time when interpersonal communication is changing rapidly, failures to exchange clinically relevant data, lack of completeness or simply defective information may lead to misunderstandings with severe patients’ safety fallouts. This may result in loss of trust and dismal team performances [5, 8]. We have all trained and operated in hospitals that relied on morning conferences, regular handovers, weekly multidisciplinary meetings, morbidity and mortality rounds, audit days, journal clubs, etc. just to abruptly see the backbone of our practice dismantled in few days. Digital health has been rapidly implemented with telemedicine consultations, virtual meetings, remote collection and analysis of clinical data. Although several authors had previously reported on the use of for example “Skype”, “Zoom”, “MS Teams” and many other tools in neurosurgery, we should immediately establish new standards of communication among our teams to ensure safe and secure transfer of the patients’ data without any breach of confidentiality [7, 11].

Similarly, the barriers in communication between neurosurgeons, patients and their families are also entirely new: COVID-19 positive patients are rapidly transferred to dedicated wards, and isolated from their loved ones, sometimes without even the opportunity to say them goodbye. Moreover, visitors and biomed company representatives are largely not allowed in hospitals at this time, creating additional barriers to the supply chain and limitations to the technical support needed by many healthcare professionals. Having to accept those dramatic situations and learning to keep our rational and professional attitude despite them have never been added to the healthcare algorithm before. It is very probable, that these difficulties will affect us from now on [16]. During the last few years, the hospitals that coped better with the aftermath of terror attacks in Europe are those that paid attention to providing immediate psychological support to their staff [9]. We therefore urge all our members that would feel it appropriate for safeguarding their well-being, and that of their staff, to seek psychological counselling and create the appropriate social safe-net within their hospital environment; we firmly believe that social distancing should never mean social isolation.

## Learning on the field while dealing with physical and psychological stress

Neurosurgeons as any other healthcare workers in the epicentre of the COVID-19 crisis are asked to manage unexpected clinical scenarios and newly emerged situations without relevant experience or preparation. Different countries, cultures and societal structures are posing different challenges to our members. After drastic reduction/disappearance of slots for elective surgery, neurosurgeons are facing increased psychological stress, been involved in a difficult decision making process about triaging their patients and choosing which procedures should be postponed or cancelled. This stress partly results from a lack of generally accepted orientation and guidance. Many societies are working hard to provide the adequate ethical basis for such decisions. A generally accepted ethical fundament is extraordinarily important when it comes to decisions beyond our scope of “normality”, for example in catastrophic situations where regular medical (intensive as well as neurosurgical) care cannot be provided for all patients at need. Although it is as much difficult to adopt established working routines as envisioning what will be the state of the art in neurosurgery after this outbreak, neurosurgeons all over the world are called to explore unchartered territories while protecting the safety of their patients, themselves and their families. EANS committees like the ethico-legal committee can contribute in these challenging times, for example by communicating of and assisting in analyses of these strictly ethical challenges related to rationing and to taking risk without proper protection.

EANS is taking a responsible lead in coordinating the activities of national neurosurgical societies through exchanging ideas, applying solutions and establishing supportive measures, including protocols, to relieve and help all the members, during this difficult period. For instance, a useful guide for triaging non-emergent neurosurgical procedures has been proposed, and warning letters regarding the higher risk of virus transmission during trans-sphenoidal approaches have been sent to all EANS members.

To help members navigating through the many educational tools available, we have reviewed the current literature and secondary data sources (see Table 2). Getting through this challenging situation, EANS could potentially gain priceless experience by strengthening the links across the whole medical profession and scientific societies offering mutual collaboration and support.

**Table 2:** Educational resources on Neurosurgery and COVID-19 outbreak

Source		Updated	Language	Hyperlink
General Information	Guidelines, recommendations, webinars, courses			
	World Health Organisation - WHO	Daily	Multiple translations	https://www.who.int/emergencies/diseases/novel-coronavirus-2019
	European Society of Intensive Care Medicine - ESICM	Daily	English	https://www.esicm.org/resources/coronavirus-public-health-emergency/
	American Medical Association- AMA	Daily	English	https://www.ama-assn.org/delivering-care/public-health/physicians-guide-covid-19
	American College of Surgeons - ACS	Daily	English	https://www.facs.org/covid-19
	Royal College of Surgeons of England - RCS Eng	Weekly	English	https://www.rcseng.ac.uk/coronavirus/
	Robert Koch Institut	Daily	German and English	https://www.rki.de/DE/Content/InfAZ/N/Neuartiges_Coronavirus/nCoV.html
	Centers for Disease Control and Prevention - CDC	Daily	English	https://www.cdc.gov/coronavirus/2019-nCoV/index.html
	High quality research papers			
	Lancet	Daily	English	https://www.thelancet.com/coronavirus
	JAMA	Daily	English	https://jamanetwork.com/journals/jama/pages/coronavirus-alert
	British Medical Journal - BMJ	Daily	English	https://www.bmj.com/coronavirus
	New England Journal of Medicine - NEJM	Daily	English	https://www.nejm.org/coronavirus?query=main_nav_lg
	Nature	Daily	English	https://www.nature.com/
Neurosurgery Specific Information	Recommendations, policies, best practices			
	Congress of Neurological Surgeons – CNS	Daily	English	https://www.cns.org/covid-19
	American Association of Neurological Surgeons – AANS	Daily	English	https://www.aans.org/COVID-19-Update/COVID-19-Information-Hub
	European Association for Neurosurgical Societies - EANS	Weekly	English	https://www.eans.org/page/Neuroscientific_aspects_of_Corona
	Italian Society for Neurosurgery - SiNCH	Weekly	Italian	http://www.sinch.it/news/covid-and-neurosurgery-10
	The Society of British Neurological Surgeons – SBNS	Weekly	English	https://www.sbns.org.uk/index.php/policies-and-publications/covid/
	Neuro-Anaesthesia and Critical Care Society - NACCS	Weekly	English	https://naccs.org.uk/covid-19-resource-page/

### Limitations

This position statement is primarily intended to benefit neurosurgeons by sharing tailored information in a carefully written and timely delivered message; of note, it was conceived through a bottom-up approach, where members approached the IMC in search for opportunities to echoing their unmet needs. This aspect should be kept in the highest consideration by politicians, as well as healthcare professionals holding managerial positions. A series of limitations can be highlighted. With regard to surveys, although these tools are relatively inexpensive and powerful, we recognise the inherent limitations associated with such type of qualitative research. More specifically, at present, we do not possess access to the PICO research questions that have inspired each survey; we do not have any insights on the methodology used to calculate their sample size, nor the sampling strategy adopted to ensure statistical relevance. As such, we encourage neurosurgeons and journalists to be mindful of the possible sampling and interpretation biases that might affect the generalizability of those research tools.

Furthermore, multiple factors, such as the lack of understanding of this virus, and the presence of a broad number of regulating healthcare bodies in Europe and the rest of the world, are limiting the possibility to compare and establish conclusions regarding the value of best practices. These represent significant obstacles to widely enforce protective measures, and might result in an underestimation of the threat.

Finally, the lack of scientific evidence on many other aspects such as communication and psychological support at time of COVID-19 is currently limiting our ability to define recommendations and grade their strength. Last but not the least, there is some concern that scientific data on COVID-19 could be launched too fast so that its scientific reliability may be questioned in the future.

## Conclusions

This outbreak of SARS-CoV-2 is certainly demonstrating that neurosurgeons should all prepare as teams, as well as individuals, to work under pressure with technical constraints that we could have never thought possible in modern times. To face the challenges that COVID-19 pandemic will bring upon us, and limit the related damages, we need to tackle its global perspective. Neurosurgeons are encouraged to contribute addressing the transnational dimension of this pandemic by providing feedbacks and experiences. In the coming weeks and months, among the many tasks that we skilfully perform daily, we should master the art of reacting as one system and not just as excellent local singularities. Neurosurgeons who still fight and suffer on their own should take the chance to join our community.

## References

[1] Bonvehí PE, Temporiti ER (2018). Transmission and control of respiratory viral infections in the healthcare setting. Curr Treat Options Infect Dis.

[2] Bruneau, M. & EANS Skull Base Section (2020) Precautions for endoscopic transnasal skull base surgery during COVID-19 pandemic. https://cdn.ymaws.com/www.eans.org/resource/resmgr/documents/corona/eans_skull_base_section_news.pdf

[3] Dreher M, Kersten A, Bickenbach J, Balfanz P, Hartmann B, Cornelissen C, Daher A, Stöhr R, Kleines M, Lemmen SW, Brokmann JC, Müller T, Müller-Wieland D, Marx G, Marx N (2020). The characteristics of 50 hospitalized COVID-19 patients with and without ARDS. Dtsch Arztebl Int.

[4] EANS (2020) EANS advice: triaging non-emergent neurosurgical procedures during the COVID-19 outbreak. https://cdn.ymaws.com/www.eans.org/resource/resmgr/documents/corona/eans_advice2020_corona.pdf

[5] Finn R, Prisco L, Ganau M (2020) Improving gender equality in the surgical workplace. JAMA Surg. 10.1001/jamasurg.2019.6026 10.1001/jamasurg.2019.6026 32049267

[6] Ganau L, Prisco L, Ligarotti GKI, Ambu R, Ganau M (2018) Understanding the pathological basis of neurological diseases through diagnostic platforms based on innovations in biomedical engineering: new concepts and theranostics perspectives. Medicines (Basel) 5(1). 10.3390/medicines5010022 10.3390/medicines5010022 PMC5874587 29495320

[7] Graziano F, Maugeri R, Basile L, Guli C, Giugno A, Iacopino DG (2017). WhatsAPP in neurosurgery: the best practice is in our hands. Acta Neurochir.

[8] Hartley BR, Elowitz E (2020). Barriers to the enhancement of effective communication in neurosurgery. World Neurosurg.

[9] Kyle J, Skleparis D, Mair FS, Gallacher KI (2020). What helps and hinders the provision of healthcare that minimises treatment burden and maximises patient capacity? A qualitative study of stroke health professional perspectives. BMJ Open.

[10] Long C, Xu H, Shen Q, Zhang X, Fan B, Wang C, Zeng B, Li Z, Li X, Li H (2020). Diagnosis of the coronavirus disease (COVID-19): rRT-PCR or CT?. Eur J Radiol.

[11] Schaller K (2016). Editorial re: WhatsAPP in neurosurgery: the best practice is in our hands. Acta Neurochir.

[12] Schaller K (2020, 2020) Neurosurgeons in the corona crisis: striving for remedy and redemption. A message from the president of the EANS [published online ahead of print, 2020 Mar 27]. Acta Neurochir. 10.1007/s00701-020-04306-9 10.1007/s00701-020-04306-9 PMC7100390 32221731

[13] Tan YT, Wang JW, Zhao K, Han L, Zhang HQ, Niu HQ, Shu K, Lei T (2020, 2020) Preliminary recommendations for surgical practice of neurosurgery department in the central epidemic area of 2019 coronavirus infection. Curr Med Sci. 10.1007/s11596-020-2173-5 10.1007/s11596-020-2173-5 PMC7100508 32219625

[14] To KK, Tsang OT, Leung WS, Tam AR, Wu TC, Lung DC, Yip CC, Cai JP, Chan JM, Chik TS, Lau DP, Choi CY, Chen LL, Chan WM, Chan KH, Ip JD, Ng AC, Poon RW, Luo CT, Cheng VC, Chan JF, Hung IF, Chen Z, Chen H, Yuen KY (2020) Temporal profiles of viral load in posterior oropharyngeal saliva samples and serum antibody responses during infection by SARS-CoV-2: an observational cohort study. Lancet Infect Dis. 10.1016/S1473-3099(20)30196-1

[15] WHO (2020) Coronavirus disease 2019 (COVID-19) Situation Report 2020 Mar 26. https://www.who.int/docs/default-source/coronaviruse/situation-reports/20200326-sitrep-66-covid-19.pdf?sfvrsn=81b94e61_2

[16] Xiao H, Zhang Y, Kong D, Li S, Yang N (2020). The effects of social support on sleep quality of medical staff treating patients with coronavirus disease 2019 (COVID-19) in January and February 2020 in China. Med Sci Monit.

[17] Zoia C, Bongetta D, Veiceschi P, Cenzato M, Di Meco F, Locatelli D, Boeris D, Fontanella M (2020) Neurosurgery during the COVID-19 pandemic/update from Lombardy, northern Italy. Acta Neurochir. 10.1007/s00701-020-04305-w 10.1007/s00701-020-04305-w PMC7103098 32222820

